# The role of K63‐linked polyubiquitination in cardiac hypertrophy

**DOI:** 10.1111/jcmm.13669

**Published:** 2018-08-13

**Authors:** Kaowen Yan, Murugavel Ponnusamy, Ying Xin, Qi Wang, Peifeng Li, Kun Wang

**Affiliations:** ^1^ Institute for Translational Medicine Qingdao University Qingdao China; ^2^ The Affiliated Hospital of Qingdao University Qingdao China

**Keywords:** AKT, cardiac hypertrophy, NF‐κB, phosphorylation, protein kinase, TRAF6, tumorigenesis, ubiquitination

## Abstract

Ubiquitination, also known as ubiquitylation, is a vital post‐translational modification of proteins that play a crucial role in the multiple biological processes including cell growth, proliferation and apoptosis. K63‐linked ubiquitination is one of the vital post‐translational modifications of proteins that are involved in the activation of protein kinases and protein trafficking during cell survival and proliferation. It also contributes to the development of various disorders including cancer, neurodegeneration and cardiac hypertrophy. In this review, we summarize the role of K63‐linked ubiquitination signalling in protein kinase activation and its implications in cardiac hypertrophy. We have also provided our perspectives on therapeutically targeting K63‐linked ubiquitination in downstream effector molecules of growth factor receptors for the treatment of cardiac hypertrophy.

## 1 INTRODUCTION

**Figure 1:**
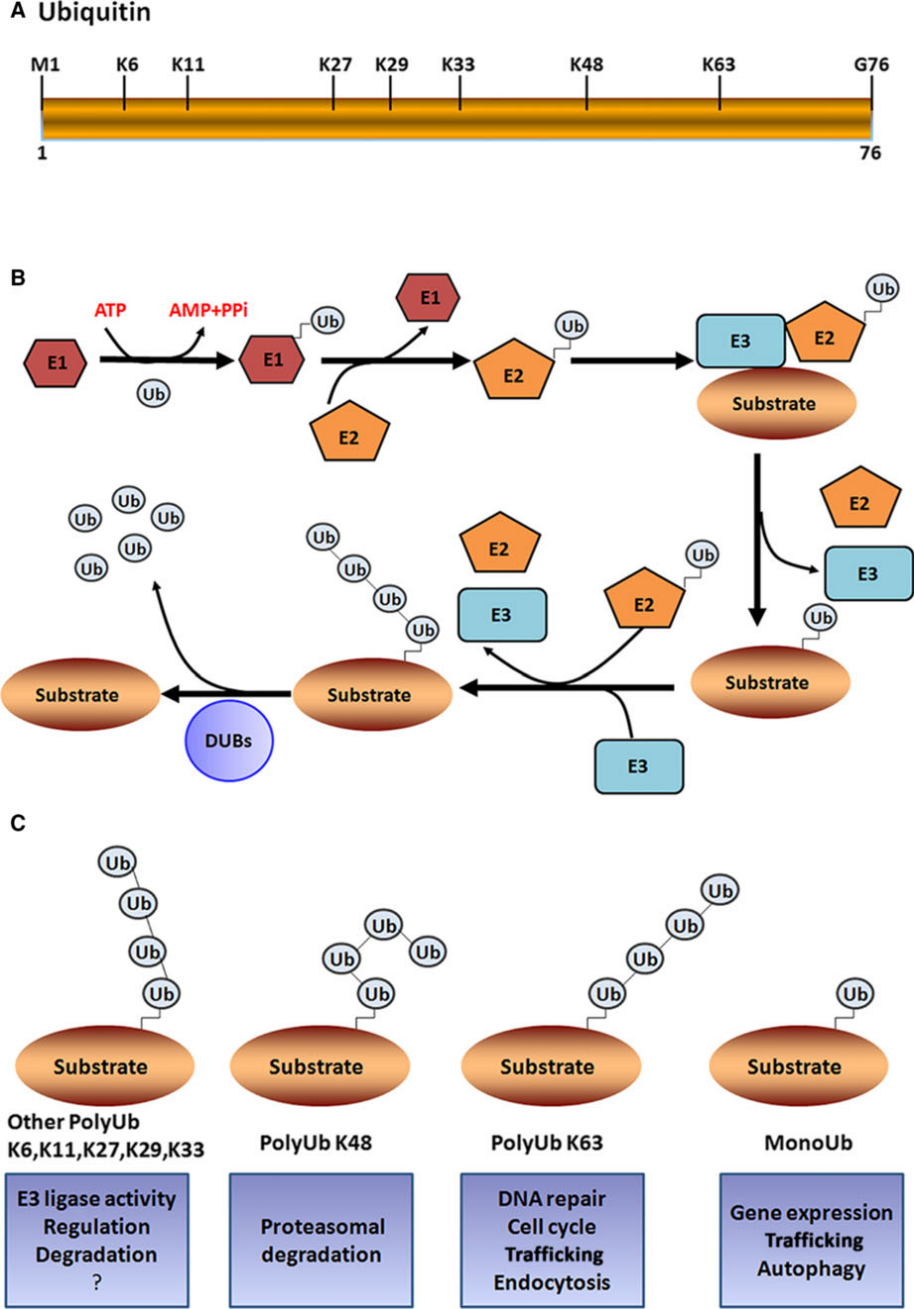
Ubiquitin and Ubiquitin modifications. A, Ubiquitin is a protein with 76 aa residues, which is highly conserved across species. It possesses 7 internal lysine residues (K6, K11, K27, K29, K33, K48 and K63) in the ubiquitin, which have been identified to be utilized for the formation of ubiquitination chains. B, The schematic representation of the ubiquitination cascade. The ubiquitin is covalently coupled with ubiquitin‐activating (E1) and then transferred to ubiquitin‐conjugating enzyme (E2), Finally, the ubiquitin ligase (E3) specifically catalyses the ubiquitination of target protein. And DUBs specifically remove ubiquitin chains from their protein substrates. C, The schematic representation of the different types of ubiquitin chains and ubiquitin signals. The question mark indicates that the roles of ubiquitin chains are largely unclear

Ubiquitin (Ub) is a protein with 76‐amino acids, which is a fundamental unit of ubiquitylation process (Figure 1A). Ubiquitination is one of the post‐translational modification processes that promote proteostatic processes by covalently attaching Ub to targeted proteins and regulating their activities and levels. In this process, Ub is specifically attached to the lysine (lys; K) residues on target proteins in a precisely timed manner through a cascade enzyme systems composed of ubiquitin‐activating enzyme (E1), ubiquitin‐conjugating enzyme (E2) and ubiquitin ligase (E3).^1^ As a first step, Ub is activated by E1 in an ATP‐dependent manner and it forms a complex with E1 enzyme through a thioester bond. Then, the activated Ub is transferred to the cysteine residue in the active site of E2. In the final step, E3 ligase is involved in the transfer of Ub from the E2 to a specific lysine residue of the substrate protein^2^, ^3^ (Figure 1B). As for phosphorylation, ubiquitination is a reversible process in which the attached Ub is removed from the target proteins by deubiquitylation enzymes (DUBs; Figure 1B). There are different types of Ub system‐dependent post‐translational modifications in proteins that diversely alter the fate of target proteins.^4^ The best‐studied ubiquitination is k48‐linked polyubiquitination, which primarily leads to proteasomal degradation.^5^ Apart from this, lysine 6‐, 11‐, 27‐ and 29‐linked polyubiquitination promotes the degradation of proteins in a 26S proteasome apparatus‐dependent manner. While K63‐linked ubiquitination of proteins contributes to various essential cellular activities including signal transduction, protein trafficking, protein‐protein interaction and DNA damage response.^6^, ^7^ Recent evidence suggests that K63‐linked ubiquitination is functionally important for various biological functions including cell cycle progression, immune response, autophagy and neural cell functions.^8^, ^9^, ^10^ Thus, the post‐translational modifications of protein by k63‐linked polyubiquitination are implicated in a wide range of cellular functions. In this review, we have focused on the role of K63‐linked ubiquitination in the cardiac hypertrophy. The exploration of the indepth mechanism by which K63‐linked ubiquitination regulates cell proliferation, apoptosis and survival could provide a new effective therapeutic strategy for the pathological cardiac hypertrophy‐associated heart dysfunction.

## 2 UBIQUITIN MODIFICATIONS

Ub is evolutionarily conserved across different species that specifically attaches to the lysine residues of targeted proteins through the sequential action of E1, E2 and E3 enzymes. The human genome encodes 2 E1 enzymes, about 50 E2 enzymes and more than 600 E3 ligating enzymes.^11^ The ubiquitination modification occurs with spatial, temporal and substrate specificity. The E2‐conjugating system determines the type of ubiquitination modification on targeted proteins, and the substrate specificity is determined by E3 enzyme. Ub contains 7 internal lysine residues (K6, K11, K27, K29, K33, K48 and K63), which is utilized for the formation of different type of polyubiquitin chains linkage (Figure 1C). The vast majority of E2‐conjugating enzymes trigger K48‐linked ubiquitination, which is a typical signal for the proteasomal degradation of substrate proteins.^2^ However, the conjugation of Ub with target substrates is not limited to the Ub‐proteasome pathway. The K63‐linked Ub chain presumably serves as a platform for various signalling pathways, and it plays a major role in the development of cancer as well as cardiac hypertrophy.

Ub modifications are classified into 3 types (monoubiquitination, multiubiquitination or polyubiquitination) according to the length and architecture of Ub chains formed in substrates.^12^ Monoubiquitination is the process of attachment of a single Ub molecule to 1 Lys residue in the target protein. It serves as a signal for the regulation of endocytosis, lysosomal targeting, meiosis and chromatin remodelling. Multiubiquitination is a process of the attachment of single Ub molecule to several Lys residues at different positions in the target protein. This type of ubiquitination contributes to the recognition of signals for the ATP‐dependent breakdown of substrate proteins by the 26S proteasome pathway. In polyubiquitination, a chain of Ub molecules is attached to a single Lys residue in the target protein, which mainly takes part in the proteasomal degradation, protein trafficking, spindle assembly during cell cycle and DNA repair^3^ (Figure 1C). In particular, the misfolded proteins are removed by the Ub‐proteasome system to maintain the cellular environment and biological events such as cellular proliferation, apoptosis and survival.

E3 ligases classically fall into 2 categories based on their structural domains: E3 ligases with HECT (homologous with E6‐associated protein C terminus) domain and E3 ligases with RING (really interesting new gene) or U‐box and PHD domain. There are more than 600 RING finger‐containing E3 ligases and >30 HECT domain‐containing ligases exist in the human. The HECT E3 ligases transiently bind with Ub by forming a thioester intermediate, before Ub transferred to substrate, but RING ligases act as scaffold, and it promotes the direct transfer of Ub to substrate by bringing the E2‐bound Ub close to substrate.^13^ The proteomic studies have shown that all types of polyubiquitination co‐exist in cells.^14^, ^15^ Lys48‐linked chains are the most abundant linkage type (often > 50% of all linkages) found in cells that are primarily involved in transfer of substrate proteins to the 26S proteasome for degradation.^16^ However, K63‐linked polyubiquitination, the second most abundant form of ubiquitylation, has various non‐proteolytic roles in cells.^8^ The K63‐linked ubiquitination acts as a molecular scaffold for protein‐protein interaction, which is important for the protein kinase signalling activation, receptor endocytosis, protein trafficking and DNA damage repair. Ub‐conjugating enzyme 13 is a major E2, which mediates K63‐specific ubiquitination with the assistance of UEV1A. The majority of E3 ligases mediate K48‐linked ubiquitination of target proteins. However, several E3 ligases such as HectH9, Mdm2, TNF receptor‐associated factor 6 (TRAF6; tumour necrosis factor receptor‐associated factor 6), cIAP1/2 (cellular inhibitor of apoptosis protein 1/2), CHIP, Parkin, UCHL1, TRAF2, ITCH and NEDD4‐2 specifically catalyse the K63‐linked ubiquitination.^17^, ^18^, ^19^ Interestingly, HectH9, Mdm2, RNF8 (ring finger protein 8) and cIAP1/2 catalyse both K63‐ and K48‐linked ubiquitination. For example, HectH9 not only ubiquitinates Myc at K63, a site critically involved in Myc transcriptional activation and the expression of a subset of Myc target genes, but also induces k48‐linked ubiquitination and proteasomal degradation of MCL‐1 and p19^Arf^.^20^ In cardiac tissue, the overexpression of TRIM8 (tripartite motif 8) exacerbates the pathological hypertrophy by triggering K63‐linked polyubiquitination of TAK1. This indicates that the development of pathological cardiac hypertrophy can be blocked by suppression of cardiac expression of TRIM8.^21^, ^22^ The activity of E3 ligase, Pellino1, is increased in the rat heart with pressure overload as well as in cultured neonatal rat cardiac fibroblasts. The suppression of Pellino1 expression can attenuate the pressure overload‐induced cardiac hypertrophy, cardiac fibrotic response and cardiac dysfunction.^23^ E3 ligase Nrdp1 enhanced I/R‐induced cardiomyocyte apoptosis by regulating ErbB3^24^ (Table 1). Interestingly, E3 ubiquitin ligases such as TRIM24 and TRIM32 show opposing effects on their target protein, dysbindin, during cardiomyocyte hypertrophy. In fact, TRIM24 promotes hypertrophic response in cardiomyocytes, while TRIM32 inhibits this response.^25^

**Table 1:** E3 ligases play important role in NF‐κB activation and cardiac hypertrophy

E3 ligases	Substrates	Function	Role in heart	References
TRAF2	NF‐κB‐inducing kinase (NIK)/MEKK1	Positively regulates the NF‐κB pathway	Promotes cardiac hypertrophy	20
TRAF6	NIK/MEKK1, IRF7	Positively regulates the NF‐κB pathway	Promotes cardiac hypertrophy	17, 18, 19
TRIM 8	Transforming growth factor beta‐activated kinase 1(TAK1)	Modulates TNFα‐and IL‐1β‐triggered NF‐κB activation	Promotes cardiac hypertrophy	21, 22
Pellino1	RIP1	Positively regulates the NF‐κB pathway	Promotes cardiac hypertrophy	23
Nrdp1	ErbB3	Suppresses downstream targets AKT, ERK1/2 and promotes activation of p38 and JNK1/2	Promotes cardiac hypertrophy	24

## 3 DEUBIQUITYLATION ENZYMES

The ubiquitination is a reversible process, and Ub chains are recycled after activation or destruction of target substrate proteins. This process is carried out by DUBs, which are a large group of enzymes with classical isopeptidases activity and more specifically targeting Ub conjugates and Ub chains. The mammalian genome encodes nearly 100 putative DUBs. DUBs are classified into 6 subfamilies according to the structure of their catalytic domain: Ub‐specific proteases (USPs), Ub C‐terminal hydrolases (UCHs), ovarian tumour‐like proteases (OTUs), JAB1/MPN/Mov34 metalloenzymes (JAMMs), Machado‐Jakob disease (MJD) proteases and a recently identified monocyte chemotactic protein‐induced protein (MCPIP) family.^16^, ^26^, ^27^, ^28^ This family of DUBs is activated depending on their specificity for the type of Ub chain conjugation attached to the substrate.^28^ The structural and functional studies found that USPs and OTUs have a characteristic catalytic core, which recognize and remove either Lys48‐ or Lys63‐linked polyubiquitin chains. For instance, Ub‐specific protease‐14 (USP14) negatively regulates the activity of proteasomes by removing Lys48‐linked Ub chains, whereas cylindromatosis tumour suppressor (CYLD) only acts on lysine 63 linkage‐specific Ub polymers.^29^ For example, CYLD attenuates TAK1 signalling by removing K63‐linked polyubiquitin chain of TAK1. This deubiquitylation process blocks TAK1‐mediated activation of the JNK‐p38 cascades, which are critical players in non‐alcoholic steatohepatitis.^30^ Some members of OTU family including OTUB1 and A20 remove Lys48‐linked Ub chains by hydrolysis, while other members of this family, such as TrABID and OTUD5, hydrolyse Lys63 linkage‐specific Ub chains.^31^, ^32^ Cezanne and Cezanne2, the 2 known DUBs specific for Lys11‐linked polyUb, regulate the NF‐κB signalling and inflammation.^33^ On the other hand, JAMMs, a family of DUBs with zinc metalloprotease activity, share the specificity for Lys63‐linked polyubiquitin.^34^, ^35^ The Ub editing activity of Josephin ATXN3 is restricted specifically to K63‐linked Ub chains and mixed‐linkage Ub chains.^36^ Given the fact that DUBs regulate the turnover rate, activation, recycling and localization of multiple proteins, they are central players in governing the signalling pathways and cell homeostasis. Due to their role in proteostasis, they have fundamental role in both normal and pathogenic cellular processes.^37^, ^38^, ^39^ Most strikingly, recent evidence indicates that the dysregulation of DUB function is closely associated with several diseases, including cancer and heart diseases.^40^, ^41^, ^42^

## 4 K63‐UBIQUITINATION PLAYS AN IMPORTANT ROLE IN NF‐κB ACTIVATION AND PATHOLOGICAL CARDIAC HYPERTROPHY

**Figure 2:**
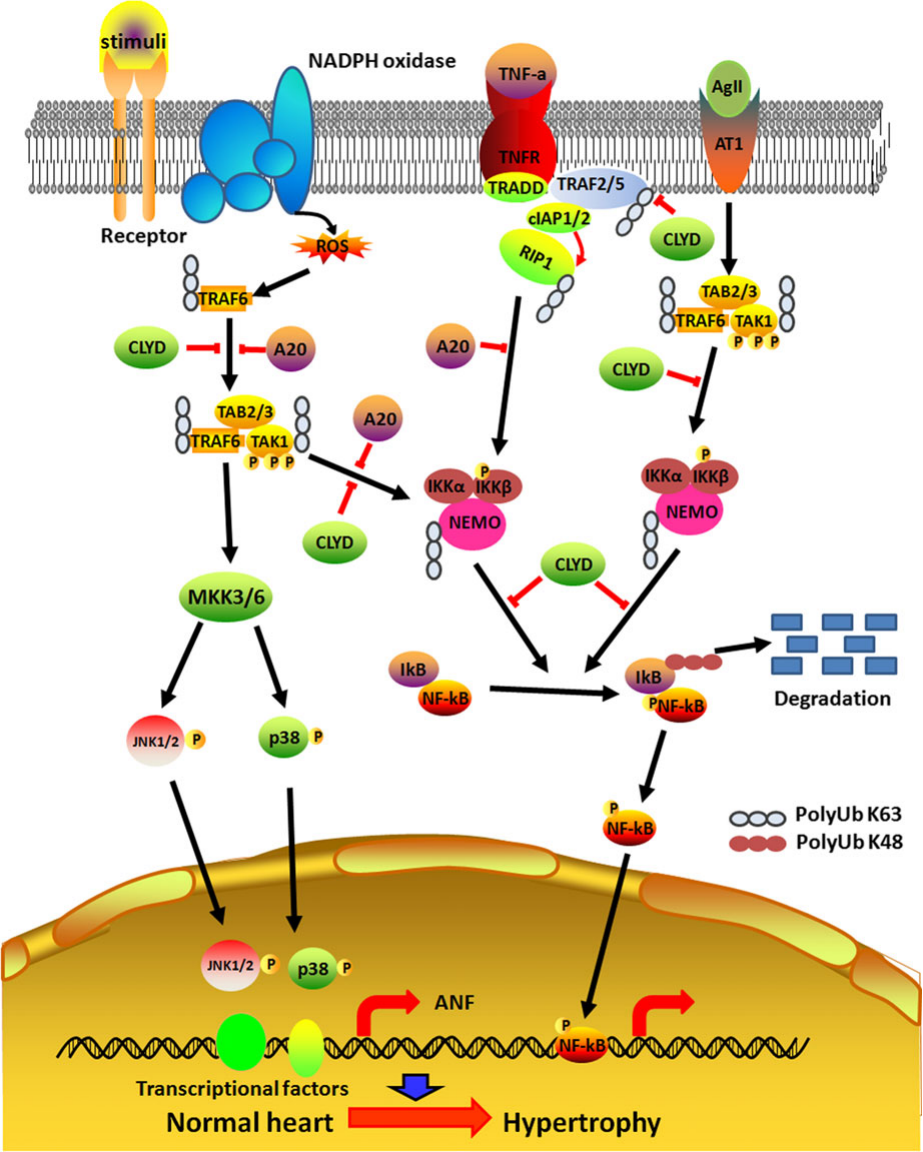
A schematic diagram of the molecular mechanisms underlying K63‐linked polyubiquitination and cardiac hypertrophy. In TNF‐α pathway, TNFR recruits its adaptor protein TRADD in response to binding of TNF‐α. TRADD further recruits TRAF2, TRAF5 and RIP1 to the receptor complex. TRAF2/5‐mediated K63 polyubiquitination of RIP1 further recruits TAB2 and TAK1 in the cytoplasm, which activates TAK1 by phosphorylation. The activated RIP1‐TAK1‐TAB2 complex subsequently promotes activation of MEKKs, which in turn activates the MAP kinase kinases, culminating in MAPK activation (JNK, ERK and p38), and activates p38 and JNK. These terminal kinases then proceed to phosphorylate transcription factors within the nucleus, as well as other regulatory proteins in the nucleus. And also K63 polyubiquitination chains of NEMO increases the activity of IKK complex which leads to IκBα‐dependent activation of NF‐κB pathway. The TNF‐α pathway is regulated by deubiquitinases A20 and CYLD

Heart failure is one of the leading causes of death worldwide, and cardiac hypertrophy is a major risk factor for the development of heart failure.^43^, ^44^ Emerging evidence suggests that K63‐linked ubiquitination plays a crucial role in the regulation of pathways such as NF‐κB, which is implicated in the development and progression of cardiac hypertrophy.^45^ NF‐κB is a ubiquitous inducible transcription factor, which can activate expression of groups of genes involved in immune response, inflammation, cell survival, apoptosis or cell growth that depends on the stimuli and extracellular factors.^46^ NF‐κB‐inducing kinase (NIK), a key molecule of non‐canonical NF‐κB signalling pathway, phosphorylates IκB kinases (IKKs), which consist of 3 subunits namely IKKα kinase, IKKβ kinase and IKKγ (a regulatory subunit). The increased activity of NF‐κB signalling contributes to hypertrophic responses.^47^, ^48^ For example, the activation of NF‐κB is necessary for the myotrophin‐induced cardiac hypertrophy in cardiomyocytes.^49^ The cardiac hypertrophic agonists such as ANG II can increase the expression and activity of NF‐κB.^50^ The cell surface receptors such as TNF receptor(TNFR), IL‐1R, Toll‐like receptor (TLR) and CD40 act as upstream activators of NF‐κB pathways and their activation by ligands leads to the recruitment of E3 ligases such as TRAF2 and TRAF6 to the receptors.^45^ Myeloid differentiation factor 88(MyD88) acts as an adaptor molecule for Toll‐like receptors (TLRs) and interleukin (IL)‐1 receptor‐dependent inflammatory signalling. K63‐linked polyubiquitination of MyD88 is involved in the activation of MyD88‐dependent TLR signalling. However, CYLD deubiquitinase system controls MyD88 activity by deubiquitination of K63‐linked polyubiquitination.^51^ For example, TNF‐α binding to the TNFR leads to the recruitment of adapter molecules TNFR‐associated death domain (TRADD) and receptor‐interacting protein 1 (RIP1) as well as the E3 Ub ligases such as TRAF2, TRAF5, cIAP1 ⁄ 2 and LUBAC.^52^ TRAF2 and TRAF6 function as E3 ligases to induce K63‐linked polyubiquitination and activation of TNFα‐dependent NF‐κB signalling. This signal is negatively regulated by CYLD‐mediated hydrolytic process of Lys63‐linked polyubiquitin. The removal of Lys63‐ubiquitylation chains from adaptor protein receptor‐interacting protein 1 (RIP1) by CYLD negatively regulates NF‐κB signalling, and this deubiquitinating event blocks the aberrant expression of survival genes in germ cells.^53^ A mass spectrometric study showed that TNFR1 complex can conjugate with Ub system by multiple polyubiquitin linkages including K48, K63, K11 and linear chains.^54^ Each linkage exhibits different responses. The K63‐linked ubiquitination of RIP1 on lysine 377 by TRAF2 is the response of inflammation stimulated by TNF.^55^, ^56^ Although the precise role of TRAF2 in TNFR signalling is currently unclear, the lipid sphingosine‐1‐phosphate (S1P) directly activates TRAF2 ligase activity and both sphingosine kinase 1 (Sphk1) and S1P are required for K63‐linked polyubiquitination of RIP1 and NF‐κB activation.^57^ In this complex, RIP1 ubiquitination might probably act as a molecular scaffold to recruit proteins bearing Ub‐binding domains (UBDs). The K63‐polyubiquitinated chain of RIP is vital for the activation NF‐κB signalling pathway. This ubiquitin chain facilitates the recruitment and formation of TAK1 kinase complex consists of TAK1 and its adaptor proteins (TAB 2/3), which is a central player in NF‐κB signalling pathway^58^ (Figure 2).

TRAF6 affects the activity of a wide range of substrates, and it acts as a crucial adaptor molecule in many signalling pathways.^59^ A recent study found that ROS‐induced activation of TRAF6 triggers its auto‐ubiquitination, which serves as an adaptor for the recruitment of TAB 2 and binding of TAK1 with TRAF6. This interaction leads to TAK1 phosphorylation and activation. A mutation study with AdTRAF6 (C70A) found that the auto‐ubiquitination of this site in TRAF6 is essential for its interaction with TAB 2 as well as for the binding and activation of TAK1. The cardiac hypertrophy stimulants, such as Ang II and phenylephrine, can increase the expression of TRAF6 in cardiomyocytes as well as in the heart tissue. TRAF6 is predominantly found in cardiomyocytes during pathological hypertrophy. The overexpression of TRAF6 aggravates Ang II‐ or pressure overload‐induced cardiac hypertrophy in animal models.^43^, ^60^ More importantly, the expression and activity of TRAF6 are increased by oxidative stress.^61^, ^62^ The activity of NADPH oxidase and reactive oxygen species (ROS) levels is dramatically increased in experimental model of cardiac pressure overload‐induced heart dysfunction and their increase is accompanied by TRAF6. The administration of apocynin (APO), a NADPH oxidase inhibitor or ROS scavenger N‐acetyl‐cysteine (NAC), can block these responses in the hypertrophic heart.^43^, ^60^ Similarly, another research group found that ROS‐mediated activation of TRAF6 promotes cardiac remodelling and the blockage of ROS production, using NAC or APO, dramatically reduces the cardiac hypertrophy and dysfunction caused by TRAF6 overexpression through attenuation of phosphorylation of TAK1. Many experimental studies confirmed that there is a direct relationship between TRAF6‐dependent K63 polyubiquitination and activation of TAK1. In transgenic mice with cardiac‐specific overexpression of TRAF6, the activity of TAK1 is increased, while it is suppressed by TRAF6 ablation, which has insignificant effects on the phosphorylation of other responders for hypertrophic stress such as TBK1, ASK1, PI3k, Ilk and Fak. It is well known that the activation of JNK1/2 and p38 is mediated by several upstream kinases in response to various hypertrophy stimuli. TRAF6 acts as an upstream activator of TAK1‐JNK1/2/p38 signalling pathway, which is associated with worsening of pathological hypertrophy. In aortic banding surgical model of cardiac failure, the overexpression of TRAF6 specifically activates JNK1/2 and p38 signalling and exacerbates the pressure overload‐induced hypertrophy and cardiac dysfunction. On the other hand, the silencing of TRAF6 reduces the phosphorylation levels of JNK1/2 and p38 in the hypertrophied heart.^63^, ^64^ These findings reveal that oxidative stress‐induced activation of ubiquitin E3 ligases such as TRAF6 plays an active role in the development of pathological cardiac hypertrophy. It is well defined that the expression of TRAF6 and the activity of TAK1‐JNK1/2/p38 cascade are increased in failing human heart. Thus, NADPH oxidase‐dependent accumulation of ROS triggers TRAF6 auto‐ubiquitination and subsequent K63‐linked ubiquitination and phosphorylation of TAK1 triggering downstream cascade of pathological hypertrophy 65, 66 (Figure 2). Together, these reports indicate that ubiquitination machineries have diverse role in cellular function and disease development, in particular, in cardiovascular system.

## 5 K63 LINKED UBIQUITINATION IN AKT ACTIVATION

**Figure 3:**
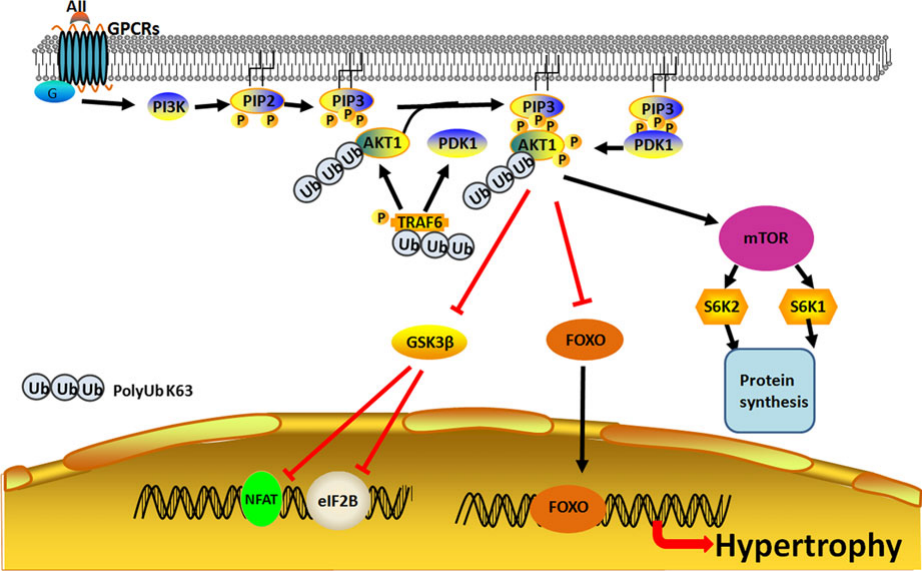
Ubiquitination regulates activation of AKT kinase. The growth factor IGF‐1 triggers K63‐linked ubiquitination of AKT and it promotes recruitment of AKT to membrane, which subsequently phosphorylated by PDK1 and mTOR. The activated AKT translocates to the nucleus and catalyses the transcription of genes associated with the heart growth and hypertrophy. E3 ligase TRAF6 contributes to Akt kinase activation by promoting K63‐linked polyubiquitination

Protein kinase B, also known as AKT, is integral part of many cells signalling cascade and they serve as a central transducer of extracellular message delivered by growth factors and cytokines to the nucleus by inducing a series of phosphorylation of intracellular proteins. AKT plays a vital role in signalling of cell growth, proliferation, differentiation, autophagy and survival.^67^, ^68^ It is well documented that AKT regulates its own activity and level by negative feedback loop. The activity of AKT in cardiac tissue is an important contributor in physiological as well as pathological cardiac hypertrophy. AKT negatively regulates glycogen synthase kinase‐3β (GSK‐3β), a ubiquitous cytoplasmic protein, by phosphorylation. Activity of GSK‐3β is catalytically active under resting conditions, but it is inhibited by AKT‐mediated phosphorylation. The active GSK‐3β has a negative effect on hypertrophic transcriptional effectors, such as GATA4, β‐catenin, c‐Myc and NFAT, and it also inhibits the translation initiation factor eIF2B.^69^, ^70^ The overexpression of GSK‐3β can blunt pathological hypertrophy caused by cardiac pressure overload.^70^ However, the aberrant activation of AKT signalling in the hypertrophic heart suppresses activity of GSK‐3β. Several reviews have covered the molecular details of K63‐linked ubiquitination and its critical role in the regulation of AKT kinase activation and cardiac hypertrophy.^71^, ^72^ In this part of review, we have focused on the regulatory function of K63‐linked polyubiquitination in protein kinase activity by taking the serine/threonine kinase AKT as an example (Figure 3). The growth factors such as insulin and insulin‐like growth factor‐1 (IGF‐1) can control the developmental and physiological growth of the heart. The binding of these ligands to IGF‐1 receptor (IGF‐1R) activates PI3K, which transduces signal to the intracellular downstream molecules.^73^ PI3K catalyses the phosphorylation of lipid phosphatidylinositol‐4,5‐bisphosphate (PIP2) and converts it to phosphatidylinositol‐3,4,5‐trisphosphate (PIP3), which activates other signalling members residing at the plasma membrane.^69^, ^73^ The activation of PI3K results in the sarcolemmal recruitment of the kinases, such as AKT and phosphoinositide‐dependent kinase‐1(PDK1).^69^ Of the 3 AKT genes, AKT1 and AKT2 are highly expressed in the heart. AKT1 is important for the proper development and size of the heart, as evident from a reduction in heart size in mice with genetic ablation of AKT1. However, AKT2 deletion does not show any change in the heart size.^74^, ^75^ Similarly, the cardiac‐specific suppression of PI3K, an immediate upstream activator of AKT, causes reduction in the size of the heart.^76^ These findings suggest that AKT signalling plays an important role in the physiological growth of the heart.

Apart from the conventional phosphorylation events, the post‐translational modification, such as ubiquitination, also involved in the regulation of activity of AKT. An E3 ligase, TRAF6, can induce K63‐linked ubiquitination of AKT, which facilitates the recruitment of AKT to plasma membrane and subsequent activation of AKT by phosphorylation at T308. TRAF6‐induced ubiquitination of AKT also plays crucial role in the translocation of AKT. The deficiency of TRAF6 in mouse embryonic fibroblast cells causes a defect in AKT localization to the membrane, which consequently leads to attenuation of cellular response to various growth factors and cytokines.^77^ A study in hepatic cells found that TAK1 autophosphorylation by the interaction of TRAF3‐TAK1 disrupts AKT‐GSK3β/FOXO1 signalling.^78^ In AKT, K8 and K14 residues within the PH (pleckstrin homology) domain are major sites of ubiquitination.^79^ This is evident from the remarkable reduction in ubiquitination level in AKT K8R and AKT K14R mutants, and this mutation blocked the recruitment of AKT to the membrane sites and its phosphorylation. This study also indicated that ubiquitination‐mediated AKT membrane recruitment does not result from PIP3 binding.^80^ Apart from this, K14 residue within the PIP3‐binding domain of AKT is required for its with PIP3, which is evident from the inability of binding of mutant (K14R) AKT with PIP3.^77^, ^81^, ^82^ The expression of TRAF2, an E3 ligase, is up‐regulated in the failing heart and its overexpression enhances cardiac hypertrophy and ventricular dysfunction by activating AKT/GSK3β signalling.^83^

## 6 DUB AND HEART

DUBs have been implicated in the regulation of cardiac hypertrophy signalling. The central components of inflammatory pathway such as NF‐κB, TAK1 and IKK can be regulated by several DUBs, which inhibit TAK1 and IKK activity by removing K63 polyubiquitin chains or inhibiting polyubiquitin chain synthesis. For example, CYLD is a DUB, which specifically cleaves K63 polyubiquitin chains.^29^, ^84^ CYLD has the capability to inhibit IL‐1, TNF and bacterial lipid polysaccharide‐induced activation of NF‐κB. However, it does not influence NIK‐dependent activation of NF‐κB, which suggests that CYLD regulates the classical pathway of activation of NF‐κB. CYLD has binding domain for TRAF2 and NEMO. The amino acid residues at 470 and 684 of NEMO are responsible for its binding with CYLD. This enables CYLD to act as an adapter protein between TRAF2 and the NEMO zinc finger, which play an important role in TNF‐induced NF‐κB signalling during embryogenesis.^85^ The deubiquitinating enzyme ubiquitin‐specific protease 4 (USP4) attenuates major hypertrophic signalling pathways, such as TAK1‐JNK and TAK1‐p38, by removing the K63‐linked polyubiquitination of TAK1. Thus, the increased expression of USP4 can suppress pathological cardiac hypertrophy. However, the expression of USP4 is decreased in failing human heart as well as in experimental animals with pathological hypertrophy.^86^ Similarly, CYLD prevents activation and recruitment of TAKl by cleaving the K63 polyubiquitin chain of TRAF2,TRAF6 and NEMO (Figure 2), which leads to inactivation of IKK and suppression of downstream of NF‐κB pathway.^84^, ^87^ Similarly, the ubiquitination of TRAF‐binding protein (TRIP) is required for the activation of TNFα‐induced NF‐κB. This signalling pathway is controlled by CYLD‐dependent removal of K63‐linked ubiquitination chains of TRIP, which leads to attenuation of TNFα‐dependent NF‐κB signalling.^88^ CYLD‐Nrf2 axis plays a key role in the cardiac remodelling. CYLD regulates cardiac maladaptive remodelling and dysfunction via interrupting the ERK, p38‐/AP‐1 and c‐Myc pathways to suppress Nrf2‐operated anti‐oxidative capacity.^89^, ^90^ Ubiquitin‐specific protease 15 (USP15) contributes to the regulation of hypertrophic responses in cardiac muscle via modulating skeletal muscle LIM protein 1 (SLIM1) at transcriptional and post‐translational levels.^91^ Another member of DUB family, A20, negatively regulates cardiac remodelling and dysfunction by inhibiting the MAPK, NF‐κB and TGF‐β signalling pathways. In addition, A20 inhibits cardiac fibrosis by blocking TAK1‐dependent activation of Smad2/3/4 signalling.^92^ A20 directly inhibits NF‐κB activity by cleaving K63‐linked ubiquitination, and it promotes K48‐linked ubiquitination on RIPl (Figure 2). This leads to attenuation of hypertrophic signalling and protects the cardiac tissue from myocardial infarction. In experimental study, the overexpression of A20 attenuated the hypertrophic stimulant such as phenylephrine‐induced development of pathological hypertrophy.^92^, ^93^ Similarly, Li et al^94^ found that cardiac‐specific overexpression of A20 significantly reduces the mortality after infarction by suppressing cardiac remodelling and improving cardiac function. Interestingly, A20 and Cezanne not only cleave polyubiquitin chains, but they also prevent the polyubiquitin chain synthesis by blocking the interaction between TRAF6 and Ubc13 and this interference can weaken the hypertrophic response in cardiomyocytes.^95^ In obesity‐induced cardiac dysfunction, A20 can prevent pathological cardiac hypertrophy by repressing the activity of its classical target TAK1 and its downstream pathways composed of NF‐κB, P38 and JNK1/2.^96^ Another member of DUB family, Ub‐specific protease 18 (USP18), also improves cardiac function, which is evident from mouse with cardiomyocyte‐specific overexpression of USP18 showing a significant increase in ventricular dilatation and ejection function as along with the reduction in cardiac hypertrophy and fibrosis. The exacerbation of cardiac remodelling in mice with USP18 deficiency further confirmed the protective role of USP18 against cardiac dysfunction caused by pathological hypertrophy.^97^ A molecular study found that USP18 inactivates TAK1 by deubiquitinating K63‐linked polyubiquitination and it subsequently suppresses the downstream NF‐κB and JNK1/2 signalling pathways, which plays critical role in NAFLD progression.^98^ USP14 also contributes to suppress the development of cardiac hypertrophy by increasing phosphorylation of GSK‐3β (Table 2).^99^ Together, these findings indicate that the most of the DUBs protect the cardiac structure and function against pathological cardiac modelling caused by various stimulus.

**Table 2:** DUBs ligases play important role in NF‐κB activation and cardiac hypertrophy

DUBs	Substrates	Function	Role in heart	References
CYLD	NEMO, TRAF2, TRAF6, TRIP	Negatively regulates the NF‐κB pathway	Induces cardiac remodelling and cardiac hypertrophy	29, 30, 53
A20	RIPl	Negatively regulates the NF‐κB pathway	Reduces cardiac remodelling and improves cardiac function	92, 93
USP15	SLIM1	Increases in protein levels of SLIM1	Positively regulates cardiac remodelling	91
USP18	TAK1	Inhibits pathological cardiac remodelling	Inhibits cardiac hypertrophy	97
USP14	NLRC5	Increase phosphorylation of GSK‐3β	Positively regulated cardiac hypertrophy	99

## 7 CONCLUSIONS

The ubiquitination and deubiquitination systems have a crucial role in the regulation of signalling pathways involved in a wide range of cellular physiological processes. The different types of polyubiquitin chains are formed within the proteins. These modifications diversely modulate the cellular function of those proteins that are depending upon the location and type of ubiquitination. For example, K48‐linked polyubiquitination signals primarily involved in the degradation of proteins. In contrast, K63‐linked polyubiquitination acts as a scaffold to recruit target proteins and facilitates protein‐protein interaction. In addition, K63‐linked polyubiquitination plays a vital role in governing the intracellular localization as well as the activities of protein kinases. The K63‐linked ubiquitination, which cooperatively works with other types of ubiquitination, is now widely recognized as indispensable for the orchestration of signalling involved in cardiac hypertrophy. Efforts have been made to specifically target ubiquitination machineries with small molecular inhibitors in order to utilize them as therapeutics.^100^, ^101^ Currently, a very few K63‐linked ubiquitinating E3 ligases are known to be involved in regulating proteins associated with hypertrophic signalling. By well defining the functional role of E3 ligases in the development or protection of cardiac hypertrophy and other cardiac diseases, E3 ubiquitin ligase may have great therapeutic potential due to their specificity to target molecules.

## CONFLICT OF INTEREST

The authors declare no conflict of interest.
